# Short-term surgical outcomes of preterm infants with necrotizing enterocolitis: A single-center experience

**DOI:** 10.1097/MD.0000000000004379

**Published:** 2016-07-29

**Authors:** Qingfeng Sheng, Zhibao Lv, Weijue Xu, Jiangbin Liu, Yibo Wu, Jingyi Shi, Zhengjun Xi

**Affiliations:** a Department of General Surgery; b Department of Neonatology; c Department of Pathology, Shanghai Children's Hospital, Shanghai Jiao Tong University, Shanghai, P.R. China.

**Keywords:** necrotizing enterocolitis, postoperative complication, surgery, survival

## Abstract

The purpose of this study was to analyze the nature of the disease, the surgical procedures, complications, and survival of preterm infants with necrotizing enterocolitis (NEC) at our institution.

Medical records of 34 preterm (gestational age <37 weeks) infants with surgical NEC were retrospectively analyzed from January 2010 to December 2014. Patients were divided into 2 groups: low birth weight (LBW, <2500 g, n = 27) and normal birth weight (NBW, ≥2500 g, n = 7).

The LBW and NBW groups differed dramatically in gestational age (31.2 ± 2.2 vs. 36.3 ± 0.5 weeks), and respiratory support (55.5% vs. 0%). The median age of NEC onset was 12 and 5 postnatal days respectively. There was an inverse association between gestational age and day of NEC onset (r = −0.470). Pneumoperitoneum, positive paracentesis, and progressive clinical deterioration were the indications for laparotomy. There was no difference in the extent of disease, in the bowel involvement, in the surgical procedures, and in the postoperative complication rates between the 2 groups. The choice of procedure has often depended upon the extent of disease (enterostomy was performed in most localized and multifocal infants, simple drainage was used in 83.3% pan-intestinal patients, *P* < 0.001). Postoperative complications occurred in 70.5% patients. The most common complications were sepsis, intestinal stricture, and short bowel syndrome. The median hospital stay was significantly longer in the LBW group (65 vs. 19 days, *P* = 0.004). The overall postoperative 180-day survival rate was 70.6% (70.4% vs. 71.5%, *P* = 0.890, log rank test). The severity of illness was the main risk factor for mortality (8.3% in localized, 18.7% in multifocal, and 100% in pan-intestinal, *P* < 0.001).

The short-term outcomes for surgical NEC are grave. The high mortality and postoperative complications in this study mandate urgent improvements in early recognition, expeditious operation, and better perioperative care.

## 1 Introduction

Necrotizing enterocolitis (NEC) remains the most common cause of gastrointestinal-associated morbidity and mortality in neonatal intensive care unit (NICU). Prematurity and low birth weight (LBW) are the most consistently agreed risk factors.^[1]^ The estimated number of live newborns in China is about 16 million per year, with 26.2% preterm (gestational age <37 weeks) and 23.6% with LBW (<2500 g).^[2]^ However, rare reports from China described the short- or long-term outcomes of patients with NEC who underwent surgical management. The purpose of this study was to evaluate the short-term results of preterm infants with surgical NEC at a single tertiary pediatric center.

## 2 Methods

A retrospectively review was carried out on 54 newborns treated surgically for NEC at Shanghai Children's Hospital from January 2010 to December 2014. Institutional Review Board was obtained from the Ethics Board of Children's Hospital, Shanghai Jiao Tong University. Written informed consent was obtained from the patient's parents. Patients who had incomplete records (n = 4), gestational age ≥37 weeks (n = 3), withdrawal of treatment (n = 6), laparotomy for intestinal stricture after medical NEC (n = 6), or major congenital heart disease (n = 1) were excluded. Therefore, the records of 34 patients were analyzed in this study. Fitzgibbons et al^[3]^ have reported that the risk and in-hospital mortality of NEC was inversely related to birth weight. So patients in present study were divided into 2 groups: LBW (<2500 g, n = 27) and normal birth weight (NBW, ≥2500 g, n = 7). The data collected included sex, gestational age, birth weight, delivery mode, obstetric and postnatal factors, age at the time of onset, age at the time of operation, radiological findings, indications for operation, the extent of the disease, distribution of intestinal involvement, operative procedures, postoperative complications, length of hospital stay, and operative survival. The extent of disease was defined as localized, multifocal, and pan-intestinal as Fasoli et al. reported.^[4]^ Follow-up was carried out in all discharged patients. Mortality was recorded within 180 days of the initial operation. Long-term outcomes such as growth and neurodevelopment are beyond the scope of this study.

Data were recorded as numeric or nominative variables. SPSS 17.0 software package (SPSS Inc, Chicago, IL) was used for statistical analysis. The differences of frequency distribution were compared using *χ*^2^ test or Fisher exact test. Survival was analyzed by Kaplan–Meier analysis (with log-rank test). Relationships between the day of NEC onset and gestation age were analyzed using Pearson correlation coefficients. Other results were expressed as mean ± standard deviation (SD). The differences between 2 groups were detected by Student *t* test or 1-way analysis of variance. Difference was considered to be significant when *P* < 0.05 (α = 0.05, 2-tailed).

## 3 Results

The demographic data of patients were shown in Table 1. There were 20 boys and 14 girls in this study with an average gestational age of 32.2 weeks. Patients in LBW group had earlier gestational age (31.2 ± 2.2 and 36.3 ± 0.5, *P* < 0.001) with 17 infants weighing <1500 g and 1 infant with extremely low birth weight (ELBW, 860 g). Maternal age, delivery mode (cesarean or vaginal), using in-vitro fertilization (IVF), incidence of multiple gestations and births, and APGAR scores (at 1 minute and 5 minutes) were similar in both groups. Complications of pregnancy occurred more in infants weighing less at time of birth (74.1% and 28.5%). However, these data did not reach statistical significance (*P* = 0.07). The status of respiratory support before or at the time of referral was reported. Preterms with lower birth weight were more likely to need ventilator support with or without surfactant administration (55.5% and 0%, *P* = 0.011).

**Table 1:**
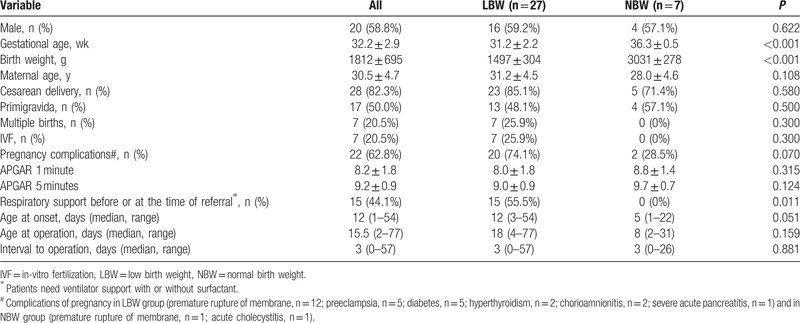
Summary of demographic and medical data.

**Figure 1:**
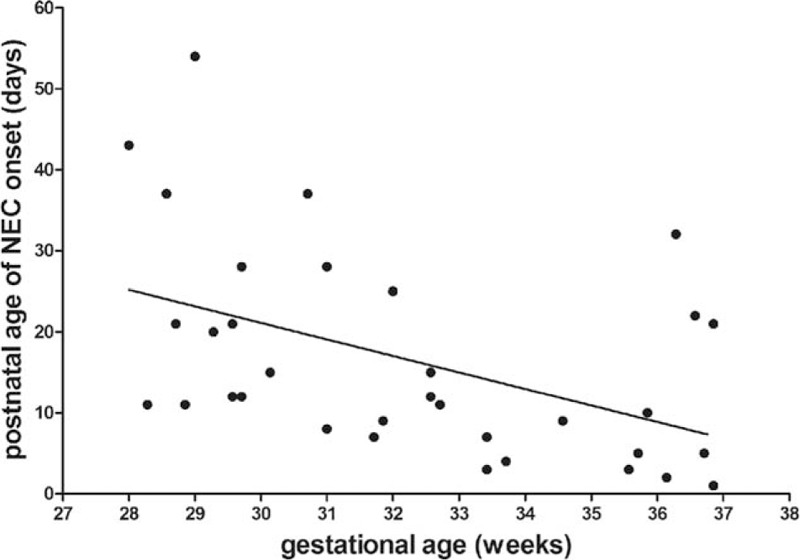
Inverse association between gestational age and postnatal age of necrotizing enterocolitis (NEC) onset by linear regression analysis (r^2^ = 0.221, *P* = 0.005).

**Figure 2:**
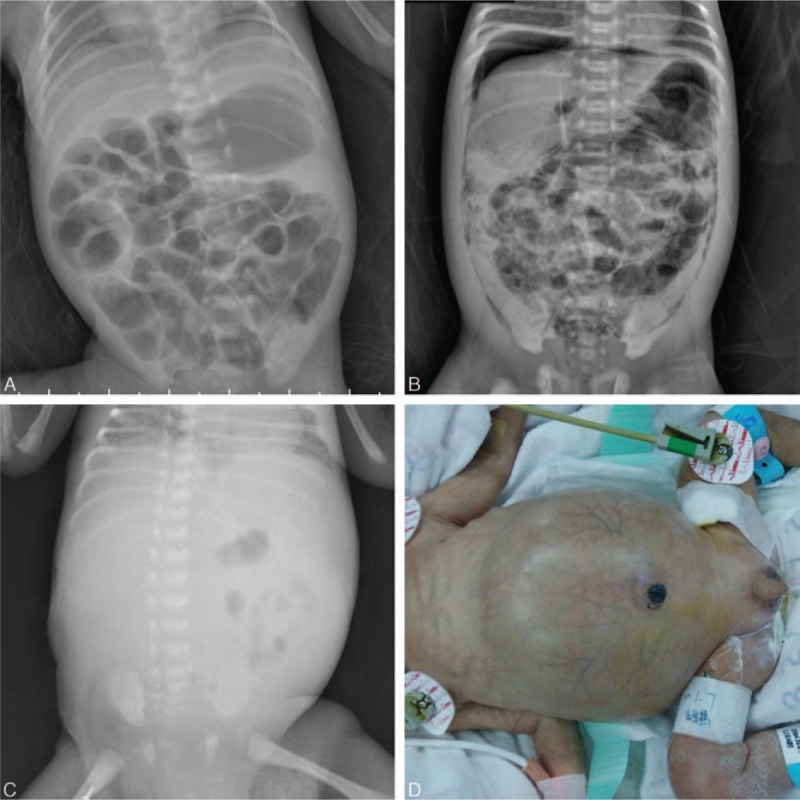
Radiographic findings of necrotizing enterocolitis. (A) Portal venous gas and pneumatosis intestinalis; (B) pneumoperitoneum and pneumatosis intestinalis; (C) gasless abdomen. Panel D shows an infant with distended abdomen with periumbilical erythema.

The median postnatal age at the onset of NEC was 12 days (range, 1–54 days). There was an inverse association between gestational age and day of NEC onset (r = −0.470, *P* = 0.005, Fig. 1). Increased gastric residuals and abdominal distension were the initial signs in great majority of cases. Laboratory findings were often nonspecific. Abdominal X-ray films (supine, cross-table lateral, or upright) remained the mainstay for diagnosis and were performed in all infants, with computed tomography (CT) scans in 9 infants. Pneumoperitoneum, portal venous gas, pneumatosis intestinalis, fixed dilated intestinal loops, and other signs such as ascites, generalized intestinal dilatation, and ileus were observed on plain radiograph or CT scan (Fig. 2). Although free intraperitoneal air was absolute indication for surgical intervention, the positive finding was only 53.8% (14/26) for plain radiograph and 77.7% (7/9) for CT. CT scan was not superior to plain radiograph (*P* = 0.262). Paracentesis was used as an adjunctive method in 17 clinical suspected cases.

**Figure 3:**
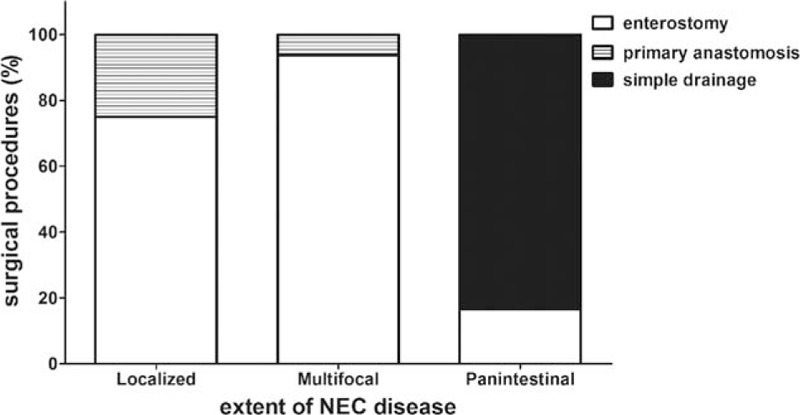
The choice of surgical procedures depends upon the extent of disease. NEC = necrotizing enterocolitis.

Indications for laparotomy in our institution included pneumoperitoneum (n = 14), positive paracentesis (n = 8), and progressive clinical deterioration (n = 12). The median postnatal age at operation was 15.5 days (range, 2–77 days) with a median interval of 3 days. Perforation was found during operation in 26 (76.5%) infants. Multifocal disease was found almost in half patients (47.1%) with 35.3% localized and 17.6% panintestinal. There were 17 infants with only small bowel involvement, combination of small and large bowel involvement in 11, and only large bowel involvement in 6. Ileum was the most common affected location (n = 28, 82.4%). As for the surgical approaches, enterostomy creation with or without necrotic bowel resection was the initial procedure in 25 patients (1 jejunostomy, 22 ileostomies, 2 transverse colostomies). Four infants required resection and primary anastomosis. The remaining 5 children underwent laparotomy and simple drainage because of panintestinal involvement with near total intestinal compromise. There was no significant difference in operative findings and surgical management between the 2 groups (Table 2). The choice of procedure has often depended upon the extent of disease as shown in Table 3 and (Fig. 3). Laparotomy with stoma formation was performed in most localized and multifocal cases. However, simple drainage was used in 83.3% panintestinal patients (*P* < 0.001). Only 2 patients underwent ileocecal valve (ICV) resection during initial operation. Patients in group LBW stayed longer in hospital when compared with NBW patients (median, 65 and 19 days, respectively, *P* = 0.004).

**Table 2:**
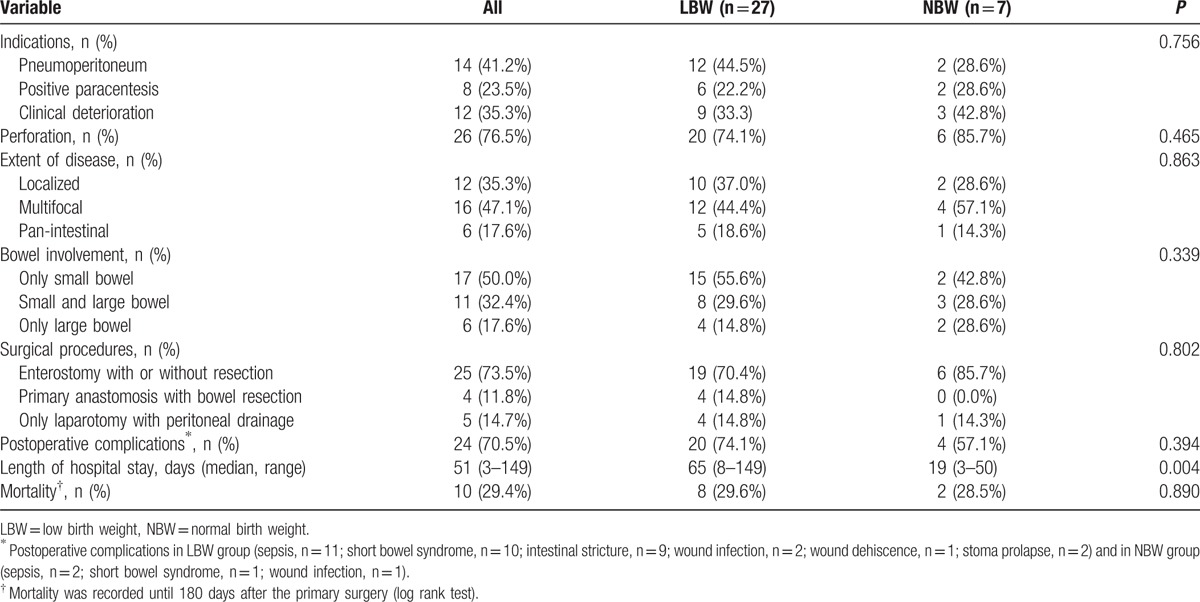
Operative data and short-term outcomes.

**Table 3:**
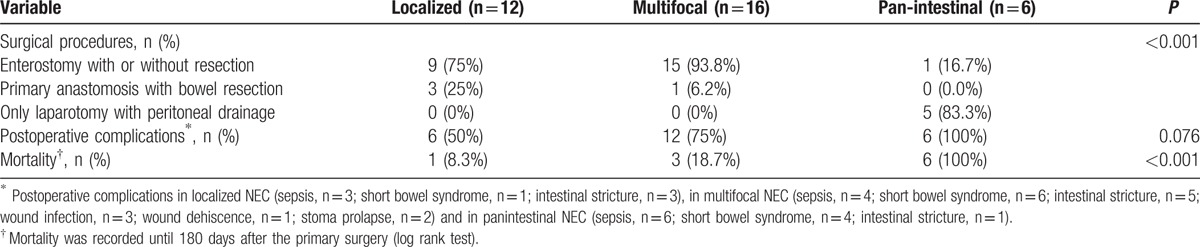
Extent of disease and short-term outcomes.

**Figure 4:**
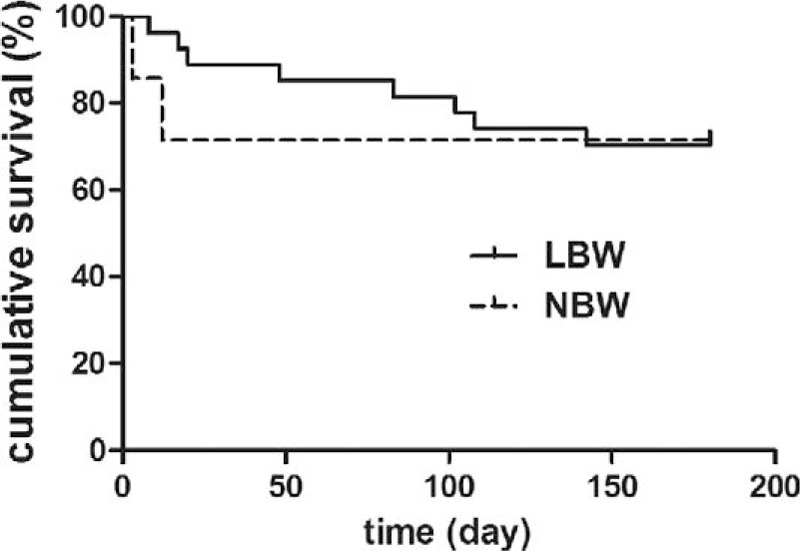
Kaplan–Meier survival analysis (group LBW, solid; group NBW, dashed). LBW = low birth weight, NBW = normal birth weight.

**Figure 5:**
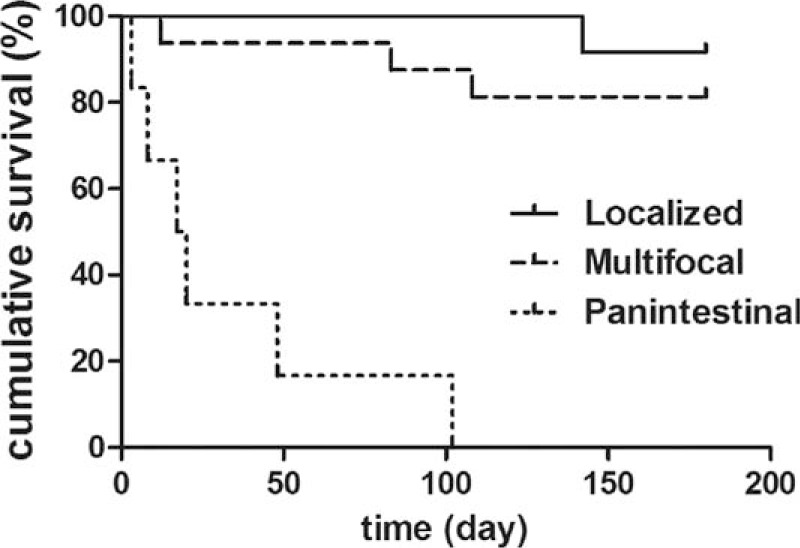
Kaplan–Meier survival analysis (localized, solid; multifocal, dashed; panintestinal, dotted).

The median follow-up period for 24 discharged patients was 2 years (range, 1–4 years). Overall postoperative 180-day survival rate was 70.6%. There was no difference in postoperative survival between the 2 groups (Table 2 and Fig. 4). The median length of survival of the 10 deaths after surgery was 4 days (range, 0–107 days). Four children died within 48 hours of surgery. There was no death occurred after stoma closure. The cause of death was primarily attributed to panintestinal NEC in 6 infants. The remaining deaths resulted from sepsis (n = 2) and severe short bowel (n = 2). Patients with less-extent disease had a significantly better operative survival (Table 3 and Fig. 5).

**Figure 6:**
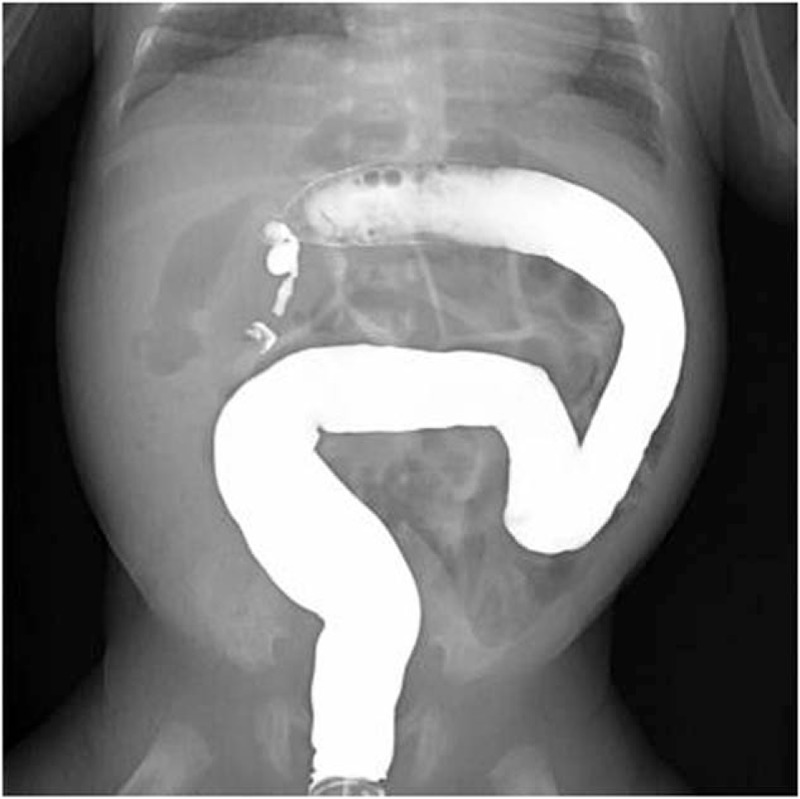
Contrast enema shows intestinal stricture in the ascending colon.

Postoperative complications occurred in 24 (70.5%) patients. The most common complications were sepsis (n = 13), short bowel syndrome (SBS, n = 11), and intestinal stricture (n = 9). The average length of residual small intestine from Treitz ligament to proximal stoma in SBS infants was 49.1 ± 7.2 cm (range, 35–60 cm). Intestinal stricture was confirmed by contrast study (Fig. 6) and operation in 9 children who underwent initial ileostomy. The average time of enterostomy reversal in this study was 128 days (range, 45–232 days) after primary laparotomy. The common affected sites were ascending colon, descending colon, and distal ileum. Seven (77.7%) patients had >1 location of stricture. Interestingly, there was no sign of stricture in any of the 4 patients who underwent primary anastomosis. Wound infections developed in three children and were managed by changing dressing. Minor stoma prolapse was noted in 2 infants, permitting manual reduction. Although adhesion was frequently encountered during stoma takedown, the need of reoperation for adhesiolysis was required in none.

## 4 Discussion

Although China has a high prevalence of prematurity, LBW newborns every year, clinical features, and outcomes of infants with surgical NEC reported in English were lacking. The present study described patient characteristics and short-term outcomes of affected infants. Demographic data revealed no sex predominance. The average gestational age (32.2 weeks) was similar to other reports from developing countries.^[5]^ The high cesarean section rate (82.3%) and less ELBW (2.9%) in this cohort were because of social factors. Of note, 25% births of the LBW group were obtained with the aid of IVF. Despite previous studies demonstrated that IVF conveyed no significant disadvantage over spontaneous conceived pregnancy, the fetal outcomes of assisted reproduction technique deserves further researches.^[6,7]^ Interestingly, there was no case in which both twin A and twin B shared the diagnosis of NEC. Infants with surgical NEC always had prematurity-associated morbidities such as bronchopulmonary dysplasia. As a result, the need for respiratory support was more frequent in LBW patients.

The high mortality (around 30%) and postoperative complications (about 70%) in this study mandate urgent improvements in early recognition, timely management (including operation), and better perioperative care. Clinical and radiographic findings are often nonspecific in the initial period. Severe signs including vital-sign instability, pneumoperitoneum, abdominal wall erythema, peritonitis, fixed abdominal mass, and gasless abdomen appear in the late phase (Fig. 2). Early recognition and diagnosis are still challenging. For many years, researchers have sought different categories of biomarkers such as cell surface antigens (neutrophil CD64), calprotectin, gut-specific proteins (intestinal fatty acid binding protein, I-FABP), and gut micorbiomes in plasma, urine, and stool samples.^[8–11]^ However, to date, there is no ideal biomarker for screening and diagnosing NEC, or predicting the severity of disease and guiding therapeutic management. The observation of inverse correlation between gestational age and postnatal age of NEC onset is consistent with previous studies.^[12,13]^ One possible explanation is the achievement of sustained and full enteral feeding at a later age in less mature infants.

Patients with definitive NEC require medical therapies including bowel rest, gastric decompression, intravenous antibiotics, parenteral nutrition, and blood product transfusion when necessary. Frequent clinical examination and serial abdominal radiography are mandatory to identify the optimal timing of surgical intervention. Recent studies reported that abdominal sonography could be used to assess bowel perfusion, viability, peristalsis, and fluid collections.^[14,15]^ Unfortunately, this noninvasive, radiation-free modality is unavailable in most neonatal centers in China. Early surgical treatment might lead to improved outcomes. The indications for operation have been described in the results section. During operation, every effort should be made to preserve as much intestinal length as possible. The option of surgical procedures largely depends upon the extent of disease and the attending surgeon's experience. Resection of necrotic bowel with enterostomy was performed in a great majority of localized and multifocal cases. But the postoperative complication rate (including stoma-related morbidity) was as high as 76% in our series. To avoid the risk of high stoma output and stoma-specific complications, resection of necrotic bowel with primary anastomosis was performed in 4 patients. Amazingly, no anastomotic leakage or intestinal stricture occurred after surgery. So resection combined with primary anastomosis is a safe, effective approach of treating NEC in selected patients. The outcomes of panintestinal NEC (“NEC totalis”) remains desperately with a mortality rate of 100%. We performed high diverting jejunostomy in 1 panintestinal case. The patient developed sepsis and SBS, and died subsequently. Other surgical alternatives including “clip and drop,” “patch, drain, and wait,” and primary peritoneal drainage have been advocated by others.^[16–19]^ However, we have no personal experience with these techniques. While deciding which operation to perform, the parents should be informed as fully as possible concerning the short- and long-term outcomes.

More than half of deaths in our study were because of “NEC totalis.” Possible reasons for high mortality and complications were as follows: difficult to early diagnosis and referral, limited resources of critical care, high rate of severe cases, delayed operation, and prolonged hospitalization. Sepsis was the most frequent complication after surgery. Prematurity, central line access, and prolonged parenteral nutrition contribute to increased incidence of septic complication.^[20,21]^ Intestinal strictures develop in 9% to 32% of patients who underwent operation.^[4,20,22,23]^ A contrast study (conventional contrast enema, distal loop enema, or both) is recommended to rule out strictures or complete obstruction before reestablishment of intestinal continuity. Koike et al^[24]^ reported that LBW infants might get benefits from enteral refeeding. And we support early stoma reversal when the infant has stabilized. The limited number of patients underwent ICV resection makes evaluation of the effect of ICV on short-term outcomes invalid. Fortunately, previous work by Fasoli et al^[4]^ indicated that duration of parenteral nutrition, length of hospital stay, recurrent NEC, and survival rate were not affected by the removal of ICV.

The limitations of the present study are as follows: retrospective design, small number of surgical NEC, some data such as maternal medication unknown, lacking long-term outcomes. Attempts to develop effective preventive strategies remain an area of ongoing research.^[25–29]^

The short-term outcomes for preterm infants with surgical NEC in this study are grave. It is crucial to recognize the optimal timing for surgical intervention. Early recognition, expeditious operation, and better perioperative care might translate into improved outcomes.

## Acknowledgements

None.
